# Regulation of Gliogenesis by *lin-32*/Atoh1 in *Caenorhabditis elegans*

**DOI:** 10.1534/g3.120.401547

**Published:** 2020-07-14

**Authors:** Albert Zhang, Kentaro Noma, Dong Yan

**Affiliations:** * Department of Molecular Genetics and Microbiology, Duke University Medical Center, Durham, NC 27710; † Graduate School of Science, Nagoya University, Nagoya, Japan 464-8602; ‡ Department of Neurobiology, Regeneration next, and Duke Institute for Brain Sciences, Duke University Medical Center, Durham, NC 27710

**Keywords:** Atoh1, glia, lin-32, neuroD1, neurog1

## Abstract

The regulation of gliogenesis is a fundamental process for nervous system development, as the appropriate glial number and identity is required for a functional nervous system. To investigate the molecular mechanisms involved in gliogenesis, we used *C. elegans* as a model and identified the function of the proneural gene *lin-32**/*Atoh1 in gliogenesis. We found that *lin-32* functions during embryonic development to negatively regulate the number of AMsh glia. The ectopic AMsh cells at least partially arise from cells originally fated to become CEPsh glia, suggesting that *lin-32* is involved in the specification of specific glial subtypes. Moreover, we show that *lin-32* acts in parallel with *cnd-1**/* NeuroD1 and *ngn-1**/* Neurog1 in negatively regulating an AMsh glia fate. Furthermore, expression of murine Atoh1 fully rescues *lin-32* mutant phenotypes, suggesting *lin-32*/Atoh1 may have a conserved role in glial specification.

In the developing nervous system, diverse sets of neuronal and glial cell types arise from common progenitors in specific spatiotemporal contexts. The mechanisms that give rise to the specific cell types are highly context-dependent, and involve the coordination of different transcription factors as well as epigenetic regulation based on both timing and position (Rowitch 2004; Temple 2001; Sugimori *et al.* 2007; Hirabayashi and Gotoh 2010). Given the vast complexity of the system and the diversity of developmental contexts, there is still much to be known about the molecular mechanisms of glial fate determination.

Proneural genes were first discovered and studied in *Drosophila*, and many show functional and sequence conservation among vertebrates and invertebrates (Jan and Jan 1994; Jiménez and Modolell 1993). Such factors including the neurogenin, atonal, NeuroD and Achaete-Scute families all have basic helix-loop-helix (bHLH) motifs and were found to play major roles regulating neurogenesis during different stages of development (Bertrand *et al.* 2002; Ross *et al.* 2003; Sugimori *et al.* 2007). At the same time, certain proneural genes such as Neurog1 and NeuroD1 have been found to play important roles in the neuron-glia fate decision, where they independently inhibit a glial fate while promoting a neuronal one (Morrow *et al.* 1999; Sun *et al.* 2001; Tomita *et al.* 2000). Here, we use the *C. elegans* glia as a model to study the molecular mechanisms regulating gliogenesis. *C. elegans* glia share lineages with neurons and show functional similarity with mammalian ones (Bacaj *et al.* 2008; Oikonomou and Shaham 2011; Ward *et al.* 1975). We show that similar to what was reported in mammalian systems, loss-of-function in *C. elegans* Neurog1 and NeuroD1 homologs *ngn-1* and *cnd*-1 give rise to additional glial cells, suggesting that *C. elegans* gliogenesis likely utilize similar mechanisms as those in mammals.

To identify additional factors involved in gliogenesis, we carried out an unbiased genetic screen and identified the role of *lin-32*/Atoh1 in glial specification. *lin-32* was previously reported to regulate the neuronal fate specification of multiple cell lineages including neurons in the male tail (Portman and Emmons 2000; Zhao and Emmons 1995; Rojo Romanos *et al.* 2017); CEPD, ADE and PDE dopaminergic neurons (Doitsidou *et al.* 2008) and the URX oxygen-sensing neurons (Rojo Romanos *et al.* 2017). Its mammalian homolog Atoh1 has also been implicated in the generation of inner ear hair cells (Bermingham *et al.* 1999) and cerebellar granule neurons (Ben-Arie *et al.* 1997), where overexpression can induce transdifferentiation of glial-like support cells into functioning hair cells in the cochlea and specify differentiation of mature cerebellar granule neurons at the expense of glial production in embryoid bodies (Izumikawa *et al.* 2005; Srivastava *et al.* 2013; Sayyid *et al.* 2019). Furthermore, Atoh1 exhibits functional conservation with *Drosophila atonal* where it also promotes a neuronal fate (Ben-Arie *et al.* 2000), while sensory precursors of the *ato* lineage generate the bulk of glia in the antenna (Jhaveri *et al.* 2000; Sen *et al.* 2005). Thus, Atoh1/*lin-32* appears to play varying roles depending on developmental context. We found that *lin-32* loss of function leads to increased numbers of certain glia cells such as AMsh and AMso glia, while having reduced numbers of other glia and neurons. Further investigation show that *lin-32* acts in early progenitor cells and in parallel with *cnd-1* and *ngn-1* in glial specification. Our results suggest that *lin-32* is involved in the regulation of glial specification across different cellular lineages. More interestingly, expression of murine Atoh1 can fully rescue *lin-32* mutant phenotypes, indicating that our findings may represent a conserved function for this gene during gliogenesis.

## Materials and methods

### C. elegans genetics

*C. elegans* strains were grown on nematode growth media (NGM) plates with *E. coli* OP50 as their food source. Animals were grown according to standard methods at 20° unless otherwise stated (Brenner 1974). Wild type worms were of the Bristol N2 strain. All transgenes, strains and DNA constructs used are described in Table S1. *yadIs46* (*Pf16f9.3*::*GFP*) was used to visualize AMsh cells while *Pttx-3*::*RFP* was used as a coinjection marker.

The recessive allele *lin-32**(**yad67**)* was isolated from a visualized EMS mutagenesis screen of over 4000 haploid genomes and was the only allele isolated in the screen with the ectopic AMsh phenotype. During backcrossing, we noticed that *yad67* was on the left arm of chromosome X, and whole genome sequencing revealed that *lin-32* was the most likely candidate in the region. The mutation was confirmed through rescue experiments.

### Cloning and constructs

All DNA expression constructs were generated using Gateway cloning technology (Invitrogen, Carlsbad, CA) and subsequently sequenced. *lin-32*, *ngn-1* and *cnd-1* cDNA were all amplified from a homemade genomic DNA pool. Promoters of *lin-32* (Forward cgccacccgattagagactag; Reverse ggttggtctgactgaaaacgacgatgtgtgag), *ngn-1* and *cnd-1* were amplified from genomic DNA using the 2kb sequence upstream of the transcription start site. Murine Atoh1 cDNA was amplified from a home made cDNA pool. In general, plasmid DNAs used in this study were injected at a concentration of 1-50ng/µL with a *Pttx-3*::*RFP* co-injection marker injected at a concentration of 50ng/µL.

### Microscopy

Representative images were acquired with a Zeiss LSM700 confocal microscope using a Plan-Apochromat 40x/1.4 objective. Worms were immobilized using 1.5% 1-phenoxy-2-propanol (TCI America, Portland, OR) in M9 buffer and mounted on 5% agar slides. 3D reconstructions were done using Zeiss Zen software as maximum intensity projections. A Zeiss Axio Imager 2 microscope equipped with Chroma HQ filters was used to score AMsh number defects. Any animal with more than the wild type AMshL and AMshR glia were scored as having the defect. Each condition represented 3 experiments of at least 50 D1 animals each that were picked at random from the culture plate unless otherwise noted, in accordance with previous literature in *C. elegans*. Cell numbers were quantified by counting the number of red nuclei labeled by *Pf16f9.3*::*mCherry*::*H2B* and confirmed by referencing the whole cell morphology labeled by *Pf16f9.3*::*GFP*.

For tracking of AMsh cell number during larval development, 10 individual L4 worms per genotype were scored under the Zeiss Axio Imager 2 microscope without 1-phenoxy-2-propanol and recovered from the agar slide. They were scored again when they reached the D1 adult stage.

### Statistical analysis

Data were analyzed using one-way ANOVA followed by Tukey’s HSD test, Chi-square test, two-tailed Student’s *t*-test, Spearman’s Rank-Order Correlation and Pearson Correlation in Graphpad Prism (Graphpad Software, La Jolla, CA).

### Data availability

Further information and requests for resources should be directed to and will be fulfilled by the Lead Contact, Dong Yan (dong.yan@duke.edu). *C. elegans* strains and plasmids generated in this study are available from the lead contact without restriction. Supplemental Figures S1 and S2 as well as Strain Table S1 are available at figshare: https://doi.org/10.25387/g3.12650363.

## Results

### Mutants of Neurog1 and NeuroD1 homologs possess additional glia cells

**Figure 1:**
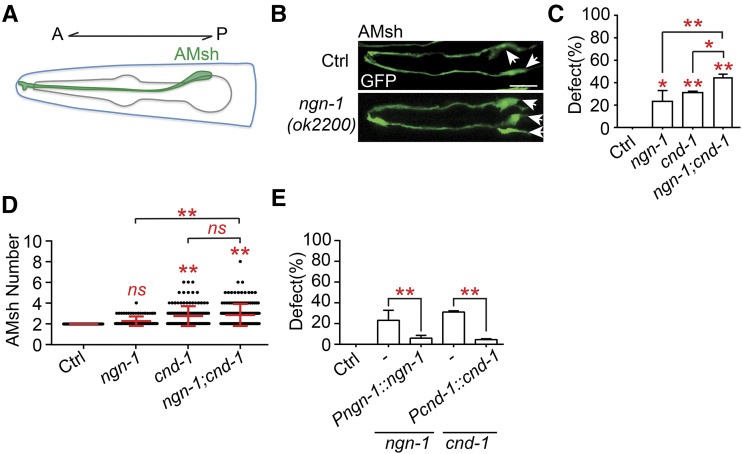
*ngn-1* and *cnd-1* regulate glial cell number (A) Schematic representation of the *C. elegans* head with an AMsh labeled in green. A represents anterior while P represents posterior. (B) Confocal images of AMsh cells during the D1 adult stage in wild type (WT) and *ngn-1**(**ok2200**)* animals expressing *Pf16f9.3*::*GFP(**yadIs46**)*. White arrows point to AMsh cell bodies. Scale bar, 10 µm. (C) The proportion of animals with additional AMsh cells labeled by *Pf16f9.3*::*GFP(**yadIs46**)* in D1 adults. Data are represented as mean ± SD. One-way ANOVA, followed by Tukey’s HSD test, *P < 0.05 **P < 0.01. *ns*, not significant. Each column represents three biological replicates of at least 50 worms each time. (D) Scatter plot of AMsh cell number in D1 adults. Each dot represents one animal, n > 80. Mean ± SD is represented in red. One-way ANOVA, followed by Tukey’s HSD test, *P < 0.05 **P < 0.01. *ns*, not significant. (E) Rescue experiments of *ngn-1**(**ok2200**)* and *cnd-1**(**gk718**)* animals using *Pngn-1*::*ngn-1* and *Pcnd-1*::*cnd-1* respectively. Data are represented as mean ± SD. One-way ANOVA, followed by Tukey’s HSD test, *P < 0.05 **P < 0.01. *ns*, not significant. Each column represents three biological replicates of at least 50 worms.

To study the molecular mechanisms underlying gliogenesis, we focused mainly on the AMsh glial cells, which are a pair of glia that ensheath the dendrites of sensory neurons in the amphid sensilla, the primary chemosensory organ (Oikonomou and Shaham 2011). AMsh glia are critical for the neurons they envelop to function, and they are easy to visualize *in vivo* (Oikonomou and Shaham 2011). To determine whether *C. elegans* shares similar mechanisms with mammals during gliogenesis, we tested the functions of the homologs of two well-studied proneural genes Neurog1 and NeuroD1 during AMsh genesis. We found that loss of function mutations of *C. elegans* Neurog1 and NeuroD1 homologs, *ngn-1* and *cnd-1*, caused approximately 20% of *ngn-1**(**ok2200**)* and 30% of *cnd-1**(**gk718**)* animals respectively to possess more than the invariant two AMsh cells observed in wild type animals when examined during the day 1 adult stage (D1). The *ngn-1**(**ok2200**);**cnd-1**(**gk718**)* double mutants had around a 45% mutant phenotype, which is significantly higher than in either of the single mutants (Figures 1A, 1B and 1C). Furthermore, *cnd-1* and *cnd-1**;**ngn-1* mutants had higher mean numbers of AMsh cells than wild type animals, while the difference was not statistically significant in *ngn-1* animals likely due to the low penetrance of phenotypes (Figure 1D). These results demonstrate that *ngn-1* and *cnd-1* function in parallel during AMsh formation, and the relatively low penetrance of phenotypes also suggest additional factors are involved in AMsh genesis as well (Figure 1C). Consistent with loss of function, expression of *ngn-1* and *cnd-1* under their own promoters strongly rescued the ectopic AMsh phenotype in their respective mutants (Figure 1E). Thus *cnd-1* and *ngn-1* are important in regulating glial specification, consistent with the role of Neurog1 and NeuroD1 in mammals (Sun *et al.* 2001; Morrow *et al.* 1999; Tomita *et al.* 2000), and supports that *C. elegans* may share common mechanisms with mammals during gliogenesis.

### A forward genetic screen reveals that loss-of-function of lin-32 results in ectopic AMsh glia

**Figure 2:**
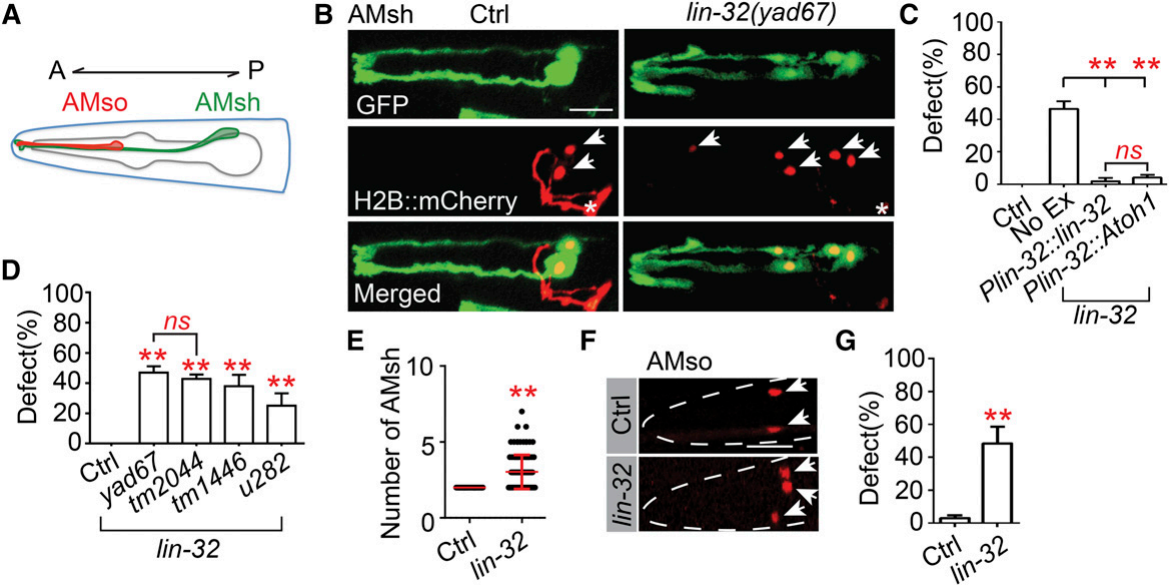
*lin-32* regulates AMsh glial cell number (A) Schematic representation of the *C. elegans* head with an AMsh cell labeled in green and an AMso labeled in red. A represents anterior while P represents posterior. (B) Confocal images of AMsh cells labeled with the *Pf16f9.3*::*GFP* reporter in green during the D1 adult stage in wild type and *lin-32**(**yad67**)* animals. The images below are of their nuclei that were labeled using the *Pf16f9.3*::*mCherry*::*H2B* reporter. Bottom row are the merged images. White arrows indicate AMsh nuclei while white asterisks indicate the AIY interneurons labeled by the *Pttx-3*::*RFP* coinjection marker. Scale bar, 10 µm. (C) Rescue experiments where *lin-32* and murine Atoh1 respectively were expressed under a *lin-32* endogenous promoter (*Plin-32*) in *lin-32**(**yad67**)* animals. Animals were quantified during the D1 adult stage. Data are represented as mean ± SD. One-way ANOVA, followed by Tukey’s HSD test, *P < 0.05 **P < 0.01. *ns*, not significant. Each column represents three biological replicates of at least 50 worms each time. (D) The proportion of animals with additional AMsh cells. *yad67*, *tm2044*, *tm1446* and *u282* are all mutant alleles of *lin-32*. Data are represented as mean ± SD. One-way ANOVA, followed by Tukey’s HSD test, *P < 0.05 **P < 0.01. *ns*, not significant. Each column represents three biological replicates of at least 50 worms each time. (E) Scatter plot of AMsh cell number in *lin-32**(**tm2044**)* D1 adults. Each dot represents one animal, n > 80. Mean ± SD is represented in red. Student’s *t*-test, *P < 0.05 **P < 0.01. *ns*, not significant. (F) Confocal images of AMso nuclei labeled using the *Pgrl-2*::*mCherry*::*H2B* reporter in D1 adult WT and *lin-32**(**yad67**)* animals. The outline of the head is marked by the dashed white line and the AMso nuclei are indicated by the white arrows. (G) The proportion of D1 adult animals with additional AMso cells. Data are represented as mean ± SD. Student’s *t*-test, *P < 0.05 **P < 0.01. *ns*, not significant. Each column represents three biological replicates of at least 50 worms each time.

Finding a function of *ngn-1* and *cnd-1* in regulating AMsh number, we decided to carry out an unbiased forward genetic screen targeting any mutants that possessed additional AMsh glia. We isolated a mutant, *yad67*, that possessed more than 2 cells labeled by the AMsh marker *Pf16f9.3* (Figures 2A and 2B). The *yad67* mutation was identified to affect the proneural gene *lin-32*, a homolog of Atoh1, consisting of a point mutation in the splice donor of its second intron (Fig. S1A). Rescue experiments confirmed *lin-32* to be the gene involved, as expression of *lin-32* under its own promoter consisting of its upstream 2kb sequence was able to fully rescue the ectopic AMsh phenotype in *yad67* mutants (Figure 2C). Interestingly, expression of murine Atoh1 under the *lin-32* promoter was also able to fully rescue the mutant phenotype, suggesting that this regulation of gliogenesis may be conserved (Figure 2C). Further testing of other alleles of *lin-32* (*tm2044*, *tm1446* and *u282*) showed that they all recapitulated the mutant AMsh phenotype observed in *yad67* animals (Figures 2D, S1A). In particular, *tm2044* is likely a null allele of *lin-32* due to it containing a deletion spanning most of the gene, including part of the basic helix-loop-helix (bHLH) domain vital for regulating transcription. Thus, the similar phenotypes and penetrance between *yad67* and *tm2044* suggest that *yad67* is likely a null allele of *lin-32* (Figures 2D, S1A). The *tm2044* allele will be used for most genetic analyses unless otherwise stated due to its similar phenotype with *yad67* and the ease of genotyping. *lin-32**(**tm2044**)* mutants possessed variable numbers of AMsh glia, with numbers ranging from 2 to 7 cells (Figure 2E). Furthermore, all the cells labeled by the *Pf16f9.3* marker were also colabeled by two other AMsh markers *Pf53f4.13* and *Pt02b11.3* (Figs. S1B-S1D), supporting the conclusion that these additional cells are AMsh glia.

To determine whether this phenotype is limited to only AMsh cells, we also examined the amphid socket (AMso) cells, which are another type of glia that come from a different cell lineage (Figure 2A) (Sulston *et al.* 1983). A similar mutant phenotype was observed in the AMso cells, where approximately 48% of *lin-32*(*yad67*) animals possessed more than the usual pair of AMso cell observed in wild type animals (Figures 2F and G).

Given the important role of *lin-32* in gliogenesis, we used AMsh glia to test other genes that have been shown to function together with *lin-32* in regulating neuron fate determination and organogenesis, including *hlh-2* which can heterodimerize with *lin-32* to regulate neuronal specification (Portman and Emmons 2000), the parallel storkhead transcription factor *ham-1**(**n1438**)* and *(**tm4595**)* (Zhu *et al.* 2014), gain of function (*n302*) and loss of function (*n941*) alleles of the potential upstream *lin-12*/Notch (Greenwald *et al.* 1983; Sundaram and Greenwald 1993; Zhong and Sternberg 2006), and a Msx homeobox homolog *vab-15**(**u781**)* found to also regulate hypodermis to neuron transformations similar to *lin-32*. None were found to have significant ectopic AMsh cell phenotypes, suggesting they may not be involved in the formation of AMsh cells (Fig. S1E). However, *hlh-2**(**tm1768**)* is not a null allele and *vab-15**(**u781**)* is not a confirmed null, so it is possible that these genes may still play a role.

### lin-32 suppresses a glial fate in different lineages during early embryogenesis

**Figure 3:**
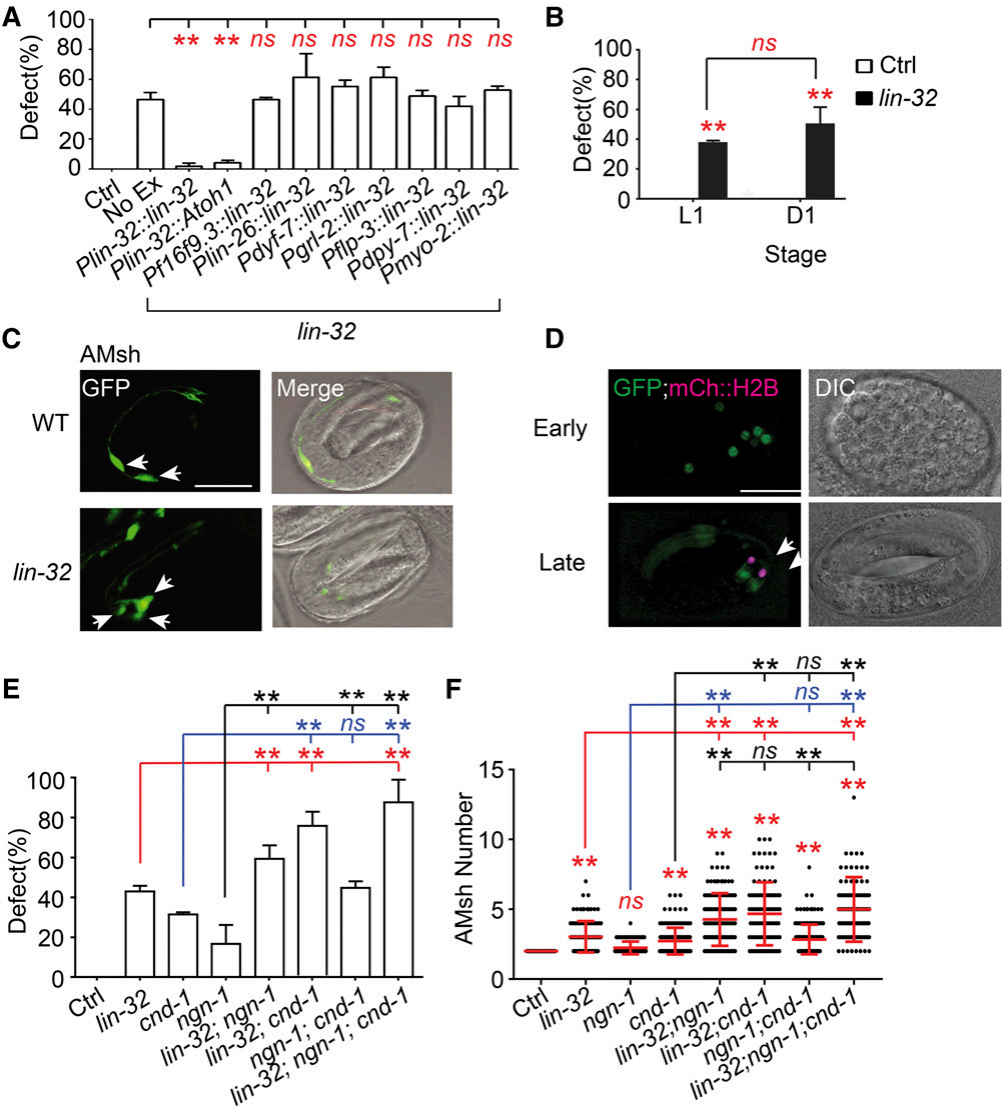
*lin-32* suppresses an AMsh fate during embryogenesis independently of *ngn-1* and *cnd-1* (A) Rescue experiments of *lin-32* driven by different promoters conducted in a WT background for the control and a *lin-32**(**yad67**)* background for the rest. Animals were quantified when they were D1 adults. Data are represented as mean ± SD. One-way ANOVA, followed by Tukey’s HSD test, *P < 0.05 **P < 0.01. *ns*, not significant. Each column represents three biological replicates of at least 50 worms each time. (B) The proportion of WT and *lin-32**(**yad67**)* animals with additional AMsh cells during the L1 larval stage and D1 adult stage. Animals were quantified when they were D1 adults. Data are represented as mean ± SD. One-way ANOVA, followed by Tukey’s HSD test, *P < 0.05 **P < 0.01. *ns*, not significant. Each column represents three biological replicates of at least 50 worms each time. (C) Confocal images of AMsh cells labeled by *Pf16f9.3*::*GFP* during late embryogenesis in WT and *lin-32**(**yad67**)* animals (left). Right column are merged with Nomarski images. White arrows indicate AMsh cell bodies. Scale bar, 10 µm. (D) Merged confocal images of a *Plin-32*::*GFP* expression reporter and a *Pf16f9.3*::*mCherry*::*H2B* AMsh reporter (left). Right column are their respective Nomarski images. White arrows indicate Amsh cell nuclei. Scale bar, 10 µm. (E) Proportion of D1 animals with additional AMsh cells in the single, double and triple mutant combinations of *lin-32**(**tm2044**)*, *ngn-1**(**ok2200**)* and *cnd-1**(**gk718**)* backgrounds. Data are represented as mean ± SD. One-way ANOVA, followed by Tukey’s HSD test, *P < 0.05 **P < 0.01. *ns*, not significant. Each column represents three biological replicates of at least 50 worms each time. (F) Scatter plot of AMsh cell number in D1 animals with single, double and triple mutant combinations of *lin-32**(**tm2044**)*, *ngn-1**(**ok2200**)* and *cnd-1**(**gk718**)* backgrounds. Each dot represents one animal, n > 80. Mean ± SD is represented in red. One-way ANOVA, followed by Tukey’s HSD test, *P < 0.05 **P < 0.01. *ns*, not significant.

As *lin-32* controls cell type determination in different neural lineages (Rojo Romanos *et al.* 2017; Zhao and Emmons 1995), we hypothesized that *lin-32* would likewise function early in development to suppress an AMsh glial fate. As expected, expression of *lin-32* in AMsh cells (*Pf16f9.3*), head neurons (*Pdyf-7*), AMso and other socket cells (*Pglr-2*), neurons including the AMsh sister URB neurons (*Pflp-3*), hypodermis (*Pdpy-7*), and pharyngeal muscles (*Pmyo-2*) were not able to rescue the additional AMsh phenotype (Figure 3A), likely due to these promoters turning on after the ectopic glial cells already arise. This is supported by transcriptomic data showing that AMsh, AMso and URB cells show little to no expression of *lin-32* (Fig. S1F) (Packer *et al.* 2019). One earlier promoter is *Plin-26*, a known regulator of glial and hypodermal cell specification that is also required for proper AMsh cell specification (Labouesse *et al.* 1996). It is expressed in several cells of the AB lineage starting from around 100 min into embryonic development including the parent cell of AMsh (Packer *et al.* 2019). However, expression of *Plin-26*::*lin-32* was not able to rescue the additional glia phenotype in *lin-32**(**yad67**)* animals (Figure 3a), suggesting that *lin-32* is required earlier or in a different set of progenitor cells.

Since many types of vertebrate glial cells have the ability to divide during development as well as after injury (Horner *et al.* 2000; Kornack and Rakic 2001; Fields and Burnstock 2006; Rusznák *et al.* 2016; Noctor *et al.* 2001), an alternative explanation is that the ectopic AMsh cells may emerge through additional proliferation. However, we found that the number of AMsh cells did not change when their number was traced in individual mutant animals from late larva to adults (Fig. S2A). Consistent with this finding, when the penetrance of the additional glial phenotype was quantified in L1 larval stage and D1 adult *lin-32**(**yad67**)* animals, it was found that there was no significant difference in the penetrance between L1 and D1 worms (Figure 3B). Further supporting this, the additional AMsh glia can already be observed late in embryogenesis, shortly after the AMsh reporter *Pf16f9.3*::*GFP* turns on (Figure 3C). Use of an integrated rescuing *lin-32*::*GFP* reporter (Yi *et al.* 2000) showed that *lin-32* is expressed in different cells before the comma stage, when the AMsh cells begin to develop, and is not detectable in the AMsh cells when the *Pf16f9.3* marker turns on (Figure 3D), nor is it present in the AMsh cells in D1 adults (Fig. S2B). These results suggest that *lin-32* functions earlier to inhibit a AMsh glial fate rather than preventing glial proliferation.

### The proneural genes lin-32, ngn-1, and cnd-1 restrict AMsh formation through independent means

Analysis of the penetrance of the additional AMsh phenotype in all the *lin-32*, *ngn-1*, and *cnd-1* double and triple mutant combinations show more severe phenotypes, which suggests that they function independently of each other to regulate AMsh cell number (Figure 3E). Furthermore, the mean number of AMsh cells increases from the single mutants to the double or triple mutants (Figure 3F). However, no significant increase in mean AMsh cell number was detected between the *lin-32**;**cnd-1* double mutant and the triple mutant despite the increase in penetrance, which could be due to a saturation of the cells that could be affected by these transcription factors (Figure 3F). On the other hand, loss of function of either *cnd-1* or *ngn-1* both lead to an increase in the number of cells expressing the P*lin-32*::*GFP* transcriptional reporter during the bean stage of embryogenesis, suggesting possible restriction of *lin-32* expression by *cnd-1* and *ngn-1* (Fig. S2C). Loss of function of either *lin-32* or *cnd-1* did not significantly affect *ngn-1* expression though (Fig. S2D). While there may be interactions between these transcription factors, they may affect different cell lineages or function during different time periods to restrict an AMsh glial fate.

### Dorsal CEPsh cells mis-differentiate into AMsh cells in lin-32 mutants

**Figure 4:**
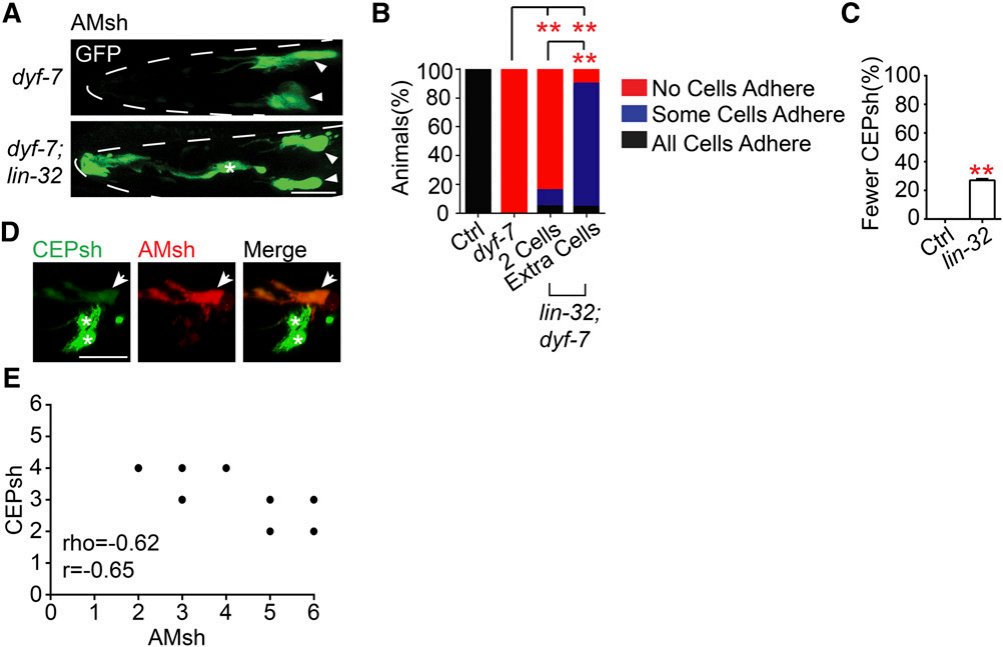
Dorsal CEPsh cells mis-differentiate into AMsh cells in *lin-32* mutants (A) Confocal images of AMsh cells labeled by *Pf16f9.3*::*GFP* in *dyf-7* and *dyf-7**;**lin-32**(**tm2044**)* mutants. Outlines of the head are demarcated by the dashed lines. White arrowheads indicate the cell bodies of AMsh cells that fail to extend their processes to the nose tip while white asterisks indicate the cell bodies of AMsh cells that extend their processes to the nose tip. Scale bar, 10 µm. (B) Proportion of WT, *dyf-7* and *dyf-7**;**lin-32* animals that either possess no AMsh cells that extend processes adhering to the nose tips, some AMsh cells that extend processes adhering to the nose tip or all AMsh cells that extend processes adhering to the nose tip. *dyf-7**;**lin-32* animals were split into two groups based on whether or not they possess more than the WT 2 am sh cells. Chi-square test, *P < 0.05 **P < 0.01. *ns*, not significant. n > 120 animals. (C) The proportion of animals with fewer CEPsh cells labeled by *Phlh-17*::*GFP* in D1 adults. Data are represented as mean ± SD. Student’s *t*-test, *P < 0.05 **P < 0.01. *ns*, not significant. Each column represents three biological replicates of at least 50 worms. (D) Confocal images of *lin-32**(**tm2044**)* animals coexpressing the CEPsh marker *Phlh-17*::*GFP* (left) and the AMsh marker *Pf16f9.3*::*mCherry* (center). Asterisks denote CEPsh cells while white arrowhead points to cell expressing both AMsh and CEPsh markers. Scale bar, 10 µm. (E) Correlation of CEPsh and AMsh cell number. Spearman’s Rank-Order Correlation (rho=-0.62) and Pearson Correlation (r=-0.65). 48 D1 adult animals were quantified.

AMsh cells and amphid neurons extend their dendritic tips through a process of retrograde extension, where the extracellular proteins DEX-1 and DYF-7 anchor the cell at the anterior end while it migrates posteriorly (Heiman and Shaham 2009). Thus, in *dyf-7* loss of function mutants, the amphid neurons and AMsh glia exhibit a process extension defect where it fails to reach the tip of the nose (Heiman and Shaham 2009). We found that all *dyf-7* mutants exhibit defects in process extension (Figure 4A). Interestingly in *lin-32**;**dyf-7* double mutants, some animals actually possess AMsh cells that properly extend their processes to the nose and no process extension defects were observed in *lin-32* mutant animals (Figure 4A). When they were further divided into animals with 2 AMsh cells and those with more, it was found that the group with more than 2 AMsh cells had over 60% more animals with some AMsh that reach the nose tip (Figure 4B). While no AMsh cells adhere in the *dyf-7* single mutants, a small percentage of AMsh cells adhere to the nose tip in *lin-32**;**dyf-7* mutants with only two AMsh cells, which are likely the original AMsh cells. It could be that loss of *lin-32* may also affect the cell expression profile of these original AMsh cells as well. It is also possible that at least some of the ectopic AMsh cells may originate from a different cell type or lineage that utilizes a different mechanism for process extension (Cebul *et al.* 2020).

Each AMsh cell arises from an asymmetric division that yields a URB neuron and an AMsh glia (Fig. S1F). Thus, it is possible that the ectopic AMsh cells in the *lin-32* mutants arise from a failure of the parent cell to divide asymmetrically or that the potential URB cell fails to take on a neuronal fate. Next, *lin-32**(**yad67**)* animals were colabeled with the *Pf16f9.3*::*GFP* AMsh marker and the *Pflp-3*::*mCherry* marker that labels 3 pairs of neurons including the URB neurons (Li *et al.* 1999). It was found that while there was a variable reduction of URB neurons in *lin-32* mutants, URB neurons alone can not account for all the ectopic AMsh cells, as some animals with more than 2 AMsh cells still have both URB cells (Fig. S2E). Furthermore, many animals possess more than 4 AMsh cells (Figure 2E), suggesting that these ectopic AMsh cells may also come from other lineages. In addition, loss of function of *unc-86*, a gene important for the terminal differentiation of URB neurons (Finney and Ruvkun 1990; Zhang *et al.* 2014), resulted in an alteration of cell identity of the neurons including the URB, but the AMsh number remained unchanged (Fig. S2F). This suggests that the inability to adopt a neuronal identity does not necessarily cause the cell to default to a glial fate, and *lin-32* likely suppresses a glial fate while independently promoting a neuronal one, similar to Neurog1 (Sun *et al.* 2001).

To determine whether the ectopic AMsh cells arise from *lin-32*-expressing cell lineages, cell death was induced in *lin-32*-expressing cells of wild type and *lin-32* mutant animals by overexpressing the apoptotic caspase CED-3 under the *lin-32* promoter (Ellis and Horvitz 1986; Shaham and Horvitz 1996). Overexpression of *Plin-32*::*ced-3* did not significantly change the number of AMsh glia in wild type animals, suggesting that these wild type AMsh glia arise from outside of *lin-32*-expressing lineages (Fig. S1F and S2G). In contrast, there was a significant reduction in AMsh number in *lin-32* mutant animals overexpressing *Plin-32*::*ced-3*, but did not fully remove all ectopic glia. This suggests that the ectopic glia at least in part arise from cells of the *lin-32* lineage, though there may also be up to roughly 50% of ectopic cells that do not arise from *lin-32*-expressing lineages (Fig. S2G).

In *lin-32* mutants, it was observed that roughly 27% of animals had 1-2 missing dorsal CEPsh glial cells (Figure 4C), which are derived from *lin-32*-expressing lineages (Murray *et al.* 2012; Packer *et al.* 2019), but the ventral two CEPsh cells that derive from cell lineages that very weakly express *lin-32* are still present in *lin-32* mutants (Fig. S1F). Thus, it is possible that ectopic AMsh cells may originate from cells originally fated to become dorsal CEPsh cells. In support of this, coexpression of the CEPsh marker *Phlh-17*::*GFP* with the AMsh marker *Pf16f9.3*::*mCherry* in *lin-32* mutants revealed that certain ectopic AMsh cells also express the CEPsh marker at early developmental stages (Figure 4D). Furthermore, when CEPsh and AMsh cell numbers were quantified in *lin-32* mutants, there was a strong negative correlation between CEPsh and AMsh cell number (rho=-0.62, r=-0.65) (Figure 4E). Thus, these ectopic AMsh cells may arise at the expense of the distantly related CEPsh cells. These results suggest that *lin-32* can function to specify glial fate among cells that come from distant cell lineages.

## Discussion

By analyzing *ngn-1*/Neurog1 and *cnd-1*/NeuroD1 mutants, we show *C. elegans* share common mechanisms with mammals in gliogenesis. We then identified the role of a proneural gene *lin-32* in regulating glial fate specification and show that *lin-32* functions in parallel to *ngn-1*/Neurog1 and *cnd-1*/NeuroD1. Furthermore, the role of *lin-32* in glial fate specification appears to be independent of its function in neuronal fate determination and likely acts in progenitor cells to restrict an AMsh cell fate. There is also potential functional conservation of *lin-32* in gliogenesis, as expression of murine Atoh1 fully rescued *lin-32* mutant phenotypes.

LIN-32 belongs to a conserved bHLH-containing proneural gene family. The first member of this family *atonal* was identified in *Drosophila* and is required for formation of the chordotonal organ and photoreceptors (Jarman *et al.* 1993; Jarman *et al.* 1994; Jarman *et al.* 1995). Furthermore, it is required for generating the majority of glia in the antenna (Jhaveri *et al.* 2000; Sen *et al.* 2005). As one of the first known transcriptional factors expressed in inner ear hair cells, Atoh1 is required for fate determination of those cells, and misexpression of Atoh1 in other cells such as the glial-like support cells is sufficient to generate hair cells (Bermingham *et al.* 1999; Ben-Arie *et al.* 2000; Zheng and Gao 2000; Kawamoto *et al.* 2003; Izumikawa *et al.* 2005; Srivastava *et al.* 2013; Sayyid *et al.* 2019). In the cerebellum, Atoh1 is required for cerebellar granule neuron formation in addition to other neurons types in the parabrachial, lateral lemniscal, and deep cerebellar nuclei, while not found to be important for gliogenesis (Ben-Arie *et al.* 1997; Wang *et al.* 2005). *C. elegans **lin-32* was first identified as an essential gene for the development of peripheral sense organs and has been shown to be important for the development of different neuronal lineages (Zhao and Emmons 1995; Portman and Emmons 2000; Doitsidou *et al.* 2008; Rojo Romanos *et al.* 2017). Interestingly, it was also found that *lin-32* activates the transcription factor *ztf-11*, which is required for specifying a postembryonic neuronal identity by repressing non-neuronal genes (Lee *et al.* 2019). Although the authors found that loss of function of *ztf-11* did not significantly affect embryonic neurogenesis, it is expressed in many cells of the AB lineage and may play a role in specification of other cell types as a downstream of *lin-32*. These studies highlighted the diverse roles of *lin-32* and its homologs in regulating neuronal and glial fate determination and sensory organ formation. Here, we uncover a function of *lin-32* in negatively regulating gliogenesis during embryonic development.

The function of Neurog1 and NeuroD1 in neuronal fate determination has been extensively investigated in different model organisms (Miyata *et al.* 1999; Morrow *et al.* 1999; Hallam *et al.* 2000; Sun *et al.* 2001; Bertrand *et al.* 2002; Ross *et al.* 2003; Hirabayashi and Gotoh 2010; Guo *et al.* 2014). Also, crossinhibitory activities of Neurog1 and Atoh1 have been shown to be essential for the specification of dorsal interneurons in mice (Gowan *et al.* 2001). Our genetic data does not support interactions between *ngn-1* and *lin-32* in gliogenesis, as *ngn-1*;*lin-32* double mutants show stronger phenotypes that are consistent with independent function. However, we do find that loss-of-function in *ngn-1* or *cnd-1* increase the number of cells that express *lin-32* in embryos, while *lin-32* does not appear to be important for *ngn-1* expression (Fig. S2C and S2D), suggesting that the regulatory interactions among *ngn-1*, *lin-32* and *cnd-1* may be more complicated than our current understanding.
